# “Semantic variant primary progressive aphasia” due to comorbidity of Lewy body disease and a previous cerebral venous infarction in the left anterior temporal lobe: A case report

**DOI:** 10.1016/j.ensci.2021.100318

**Published:** 2021-01-19

**Authors:** Kazuo Kakinuma, Wataru Narita, Toru Baba, Osamu Iizuka, Yoshiyuki Nishio, Kyoko Suzuki

**Affiliations:** a Department of Behavioral Neurology and Cognitive Neuroscience, Tohoku University Graduate School of Medicine, Sendai, Japan; b Department of Neurology, National Hospital Organization Sendai-Nishitaga Hospital, Sendai, Japan; c Department of Psychiatry & Neurology, Tokyo Metropolitan Matsuzawa Hospital, Tokyo, Japan

**Keywords:** Dementia with Lewy bodies, Primary progressive aphasia, Stroke, Semantic dementia, Treatment, Donepezil, PPA, primary progressive aphasia, svPPA, semantic variant PPA, lvPPA, logopenic variant PPA, DLB, dementia with Lewy bodies, WAB, Western Aphasia Battery, TLPA, Test of Lexical Processing in Aphasia, MoCA-J, Montreal Cognitive Assessment Japanese version, MMSE-J, Mini-Mental State Examination Japanese version, MRI, Magnetic Resonance Imaging, ^123^I-IMP SPECT, ^123^I-isopropyl-iodoamphetamine single photon emission computed tomography, DaT, ^123^I-ioflupane single photon emission computed tomography, ^123^I-MIBG, ^123^I-meta-iodobenzylguanidine

## Abstract

Primary progressive aphasia (PPA) is a neurological syndrome characterized by progressive language impairment. Various neurodegenerative disorders cause PPA. Dementia with Lewy bodies (DLB) is one known cause of PPA, and little is known about this association. Almost all published cases of PPA associated with DLB are the logopenic variant of PPA. Here, we describe the novel case of a patient with DLB presenting clinical features of the semantic variant PPA (svPPA). A 75-year-old woman was referred to our hospital with a 2-year history of progressive anomia and amnesia. Two months before admission, she had been experiencing visual hallucinations, and at the age of 60 years, she had venous infarction in the left temporal lobe, which she recovered from without any residual symptoms. Upon admission to our hospital, she displayed anomia, impaired single-word comprehension, and surface dyslexia with preserved repetition and speech production. These symptoms met the criteria for the diagnosis of svPPA. ^123^I-ioflupane single-photon emission computed tomography and ^123^I-meta-iodobenzylguanidine myocardial scintigraphy indicated DLB. Thus, she was administered donepezil, and this dramatically improved her symptoms. We hypothesize that the combination of DLB with the previous asymptomatic venous thrombosis in the left temporal lobe may have contributed to the “svPPA” in this patient. In conclusion, we show that PPA associated with DLB could be treated with donepezil, and we suggest that donepezil should be pursued as a treatment option for PPA.

## Highlights

• Primary progressive aphasia is characterized by progressive language impairment.
• We report a case of primary progressive aphasia and dementia with Lewy bodies.
• MRI showed a previously found asymptomatic lesion in the left temporal lobe.
• A cholinesterase inhibitor improved cognitive functions, including language.
• Comorbidity disrupted the recovery of temporal lobe function and impaired language.

## 1 Introduction

Primary progressive aphasia (PPA) is a neurological syndrome characterized by progressive language impairment. It is currently classified into three subtypes: the non-fluent/agrammatic variant, characterized by apraxia of speech or agrammatism; the semantic variant (svPPA), characterized by impaired naming and single-word comprehension; and the logopenic variant (lvPPA), characterized by impaired single-word retrieval and repetition of sentences and phrases [1]. Various neurodegenerative disorders cause PPA, including frontotemporal lobar degeneration, corticobasal ganglionic degeneration, progressive supranuclear palsy, and Alzheimer's disease [2]. One rare cause of PPA is dementia with Lewy bodies (DLB), and little is known about the characteristics of PPA associated with DLB. To date, almost all published cases of PPA associated with DLB are lvPPA [[3], [4], [5], [6], [7], [8]]. In this report, we describe the novel case of a patient with DLB presenting with the clinical features of svPPA.

## 2 Case presentation

A 75-year-old right-handed woman was referred to our hospital with a 2-year history of progressive anomia and amnesia. She complained of word finding difficulty and trouble spelling. Two months before admission, she had been experiencing repeated visual hallucinations of an unfamiliar person approximately once a week, and at the age of 60 years, she developed acute amnesia and headaches associated with a venous infarction in the left anterior temporal lobe. After conservative treatment, she recovered without any residual symptoms and lived independently.

Upon admission to our hospital, she showed word-finding difficulty and impaired word comprehension; however, her speech was fluent without any distortion or agrammatism. In addition, she had constipation, orthostatic hypotension, and hyposomnia, with no REM sleep behavior disorder observed. Neurological examination revealed no motor or sensory disturbances or signs of parkinsonism. On the odor stick identification test for Japanese, she scored 2 out of 12. She showed no signs of disinhibition, apathy, loss of empathy, or dietary changes. Also, the patient displayed no personality or behavioral changes.

Her performance on the neuropsychological batteries is shown in Table 1. The Western Aphasia Battery (WAB) Japanese version revealed anomia, poor single-word comprehension, superficial dyslexia, and slight impairment in writing both kanji (logogram) and Kana (phonogram), while speech production and repetition were preserved. The naming and comprehension tasks of the Test of Lexical Processing in Aphasia (TLPA) revealed two-way anomia for 23 words. Her object knowledge was preserved. Mild amnesia was evident in her daily activities and through assessment using the word recall tasks of the Montreal Cognitive Assessment Japanese version (MoCA-J) and the Mini-Mental State Examination Japanese version (MMSE-J). Copying tasks in the MoCA-J and MMSE-J were performed well; however, the Raven's Colored Progressive Matrices indicated mild visuospatial dysfunction.

**Table 1:** Results of the neuropsychological tasks.

			Baseline	Posttreatment^a^	Normal^b^
General cognitive assessment	MoCA-J		12	18	
	MMSE-J		21	26	
	Raven's Colored Progressive Matrices		22	23	29.3 ± 6.7
Language assessment	WAB	Aphasia Quotient	81.8	88	97.7 ± 3.0
		Content	8	8	9.7 ± 0.6
		Fluency	7	8	10.0 ± 0.0
		Auditory comprehension	9.2	9.4	9.8 ± 0.1
		Repetition	10	10	9.9 ± 0.3
		Naming	6.7	8.6	9.5 ± 0.6
		Reading	8.8	8.6	9.5 ± 0.8
		Writing	10	10	9.6 ± 1.0
	TLPA	Naming (200 items)	110	130	191.17 ± 6.59
		Comprehension (200 items)	167	192	199.20 ± 0.83
		Two-way anomia	23	5	

Abbreviations: MoCA-J, Montreal Cognitive Assessment Japanese version; MMSE-J, Mini-Mental State Examination Japanese version; WAB, Western Aphasia Battery; TLPA, Test of Lexical Processing in Aphasia.

a Posttreatment examinations were performed after 2 months of medical treatment with donepezil.

b Normal scores show the average ± 1 standard deviation of normal controls.

**Fig. 1:**
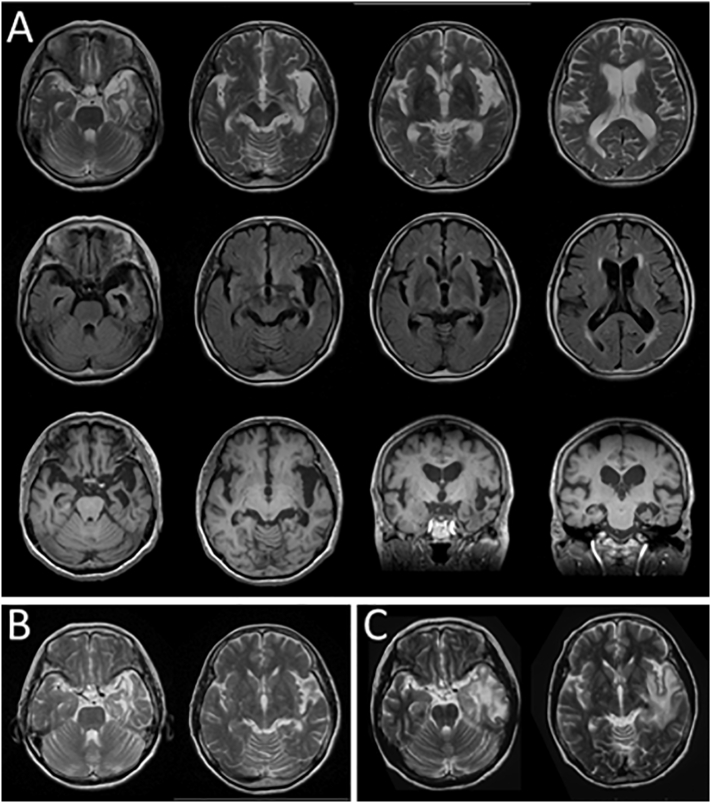
Magnetic resonance imaging data. (A) Axial T2-weighted imaging (T2WI), axial fluid-attenuated inversion-recovery images, and axial and coronal T1-weighted imaging revealed the previously diagnosed venous thrombosis in the left anterior temporal lobe. (B) The findings of T2WI were almost unchanged from those imaged 7 years before admission. (C) The T2WI images taken from when she was 60 years old showed a high-intensity area in the left temporal lobe and insula, indicating venous thrombosis with brain edema.

**Fig. 2:**
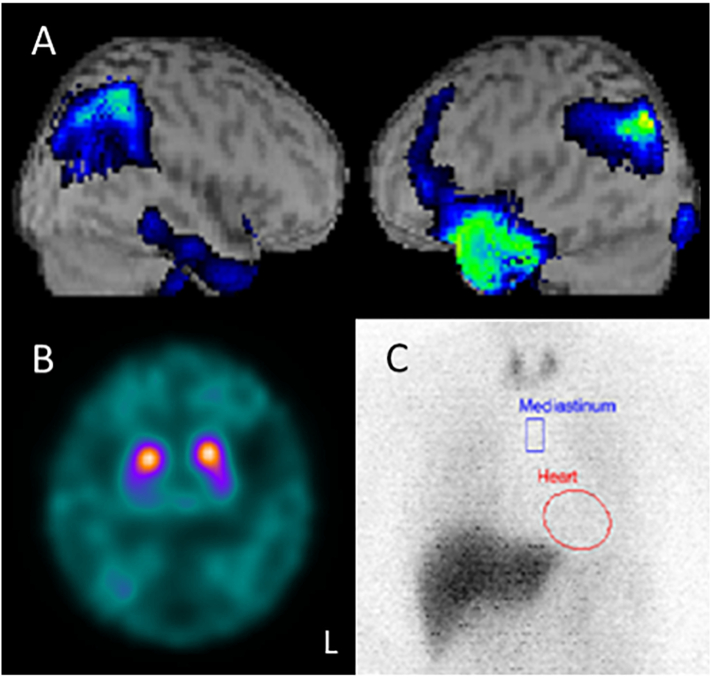
Nuclear imaging data. (A) ^123^I-isopropyl-iodoamphetamine single-photon emission computed tomography (^123^I-IMP SPECT) showed reduced cerebral blood flow in the left anterior temporal lobe and bilateral parietooccipital regions. (B) ^123^I-ioflupane single-photon emission computed tomography (DaT) scan showed reduced bilateral striatal uptake. (C) ^123^I-meta-iodobenzylguanidine (^123^I-MIBG) myocardial scintigraphy showed significantly reduced cardiac uptake.

Magnetic resonance imaging (MRI) showed diffuse mild atrophy and the previously diagnosed venous thrombosis in the left anterior temporal lobe (Fig. 1A). The MRI from 7 years before admission (Fig. 1B) showed that the venous stroke that occurred when she was 60 years old (Fig. 1C) had become obsolete, and it had remained stable for 7 years. ^123^I-isopropyl-iodoamphetamine single-photon emission computed tomography (^123^I-IMP SPECT) showed hypoperfusion in the left anterior temporal lobe and bilateral parietooccipital areas (Fig. 2A). ^123^I-ioflupane single-photon emission computed tomography (DaT) showed reduced striatal uptake bilaterally (Fig. 2B), and ^123^I-meta-iodobenzylguanidine (^123^I-MIBG) myocardial scintigraphy showed significantly reduced cardiac uptake (Fig. 2C). Based on these findings and the revised criteria for the clinical diagnosis of DLB, she was diagnosed with probable DLB [9]; thus, we started oral administration of donepezil.

Two months following treatment with donepezil, her cognitive functions, especially naming and single-word comprehension, improved (Table 1). Her scores on the MoCA-J, MMSE-J, and the naming and comprehension tasks of the TLPA were consistently higher at posttreatment than at baseline. Her two-way anomia decreased from 23 to 5 words.

Her cognitive function remained almost unchanged for 4 months after the introduction of donepezil. However, within a year, the amnesia and the visual hallucinations reoccurred. Her language functions related to naming gradually declined but remained at a higher level than before the introduction of donepezil. The impairment in single-word comprehension was mild and did not cause significant difficulties in her daily life. The articulation and fluency of her speech were preserved. Due to other physical illnesses, she was transferred to another hospital approximately a year after the initial diagnosis.

## 3 Discussion

In this case study, we show for the first time that treatment with donepezil improved cognitive functions in a patient with svPPA associated with DLB. It is interesting to note that her language symptoms corresponded with the location of her vascular lesion [10] but not the onset of the lesion, and the onset of aphasia coincided with the onset of DLB. We wondered if the left temporal venous thrombosis from 15 years ago could be associated with her aphasia that appeared 2 years ago. Based on our findings, we hypothesize that those language dysfunctions could be caused by the past venous thrombosis, and this may have been compensated by a large cerebral network, leading to improvements in language symptoms. This compensation network may have deteriorated following the pathological changes associated with DLB. Here, we showed that the administration of donepezil improved the patient's naming and comprehension, and this evidence supports our hypothesis.

There are some limitations to this study. As we did not examine language functions after the venous thrombosis 15 years ago during the chronic stage, some of her language deficits might have existed before the onset of DLB. However, the patient and her family had not noticed any difficulties associated with language until two years before admission, and there was a significant improvement in language functions following the administration of donepezil. In addition, the comorbidity of other pathological changes cannot be excluded without further neuropathological examinations.

In summary, we report a patient with svPPA associated with DLB whose language dysfunctions were resolved following the administration of donepezil. We have previously reported that donepezil improved language symptoms in a case of DLB and Alzheimer's disease with lvPPA [3]. These cases suggest that donepezil is a promising treatment option for PPA associated with DLB and associated pathologies. We propose a careful workup for screening DLB in PPA patients to avoid missing any potential therapeutic opportunities.

## Contributions

All authors contributed substantially in managing the patient and writing this case report. All authors approved the final version.

## Funding

This work was supported by a Grant-in-Aid for Scientific Research on Innovative Areas [No. 19H04890] from MEXT, Japan to KS.

## Ethical standards and consent to participate

We have obtained the patient's permission and informed consent for the publishing of her information and images. This research was approved by the Ethics committee of Tohoku University Hospital, and was under the ethical standards laid down in the 1964 Declaration of Helsinki and its later amendments.

## Declaration of Competing Interest

None.
